# Bidirectional Transcriptome Analysis of Rat Bone Marrow-Derived Mesenchymal Stem Cells and Activated Microglia in an *In Vitro* Coculture System

**DOI:** 10.1155/2018/6126413

**Published:** 2018-07-29

**Authors:** Da Yeon Lee, Moon Suk Jin, Balachandran Manavalan, Hak Kyun Kim, Jun Hyeok Song, Tae Hwan Shin, Gwang Lee

**Affiliations:** ^1^Department of Physiology and Department of Biomedical Sciences, Ajou University School of Medicine, Suwon, Republic of Korea; ^2^Ajou University School of Medicine, Suwon, Republic of Korea; ^3^Institute of Molecular Science and Technology, Ajou University, Suwon, Republic of Korea

## Abstract

Microglia contribute to the regulation of neuroinflammation and play an important role in the pathogenesis of brain diseases. Thus, regulation of neuroinflammation triggered by activated microglia in brain diseases has become a promising curative strategy. Bone marrow-derived mesenchymal stem cells (BM-MSCs) have been shown to have therapeutic effects, resulting from the regulation of inflammatory conditions in the brain. In this study, we investigated differential gene expression in rat BM-MSCs (rBM-MSCs) that were cocultured with lipopolysaccharide- (LPS-) stimulated primary rat microglia using microarray analysis and evaluated the functional relationships through Ingenuity Pathway Analysis (IPA). We also evaluated the effects of rBM-MSC on LPS-stimulated microglia using a reverse coculture system and the same conditions of the transcriptomic analysis. In the transcriptome of rBM-MSCs, 67 genes were differentially expressed, which were highly related with migration of cells, compared to control. The prediction of the gene network using IPA and experimental validation showed that LPS-stimulated primary rat microglia increase the migration of rBM-MSCs. Reversely, expression patterns of the transcriptome in LPS-stimulated primary rat microglia were changed when cocultured with rBM-MSCs. Our results showed that 65 genes were changed, which were highly related with inflammatory response, compared to absence of rBM-MSCs. In the same way with the aforementioned, the prediction of the gene network and experimental validation showed that rBM-MSCs decrease the inflammatory response of LPS-stimulated primary rat microglia. Our data indicate that LPS-stimulated microglia increase the migration of rBM-MSCs and that rBM-MSCs reduce the inflammatory activity in LPS-stimulated microglia. The results of this study show complex mechanisms underlying the interaction between rBM-MSCs and activated microglia and may be helpful for the development of stem cell-based strategies for brain diseases.

## 1. Introduction

Microglia are resident immune cells of the brain that participate in various physiological functions, such as pruning, regulating plasticity, and neurogenesis, to maintain homeostasis [1]. Multibranched resting microglia exist in a quiescent state in healthy conditions; however, upon sensing a disturbance in homeostasis, microglia become more rounded and amoeboid-shaped and increase phagocytosis and secretion of proinflammatory cytokines [2]. Activated microglia induce inflammatory environments that are related to neurological diseases such as neurodegenerative disorders, multiple sclerosis (MS), stroke, and neuropathic pain disease [3–6].

Regulation of microglia-mediated inflammation has been considered a therapeutic strategy in brain diseases. Several anti-inflammatory drugs such as glucocorticoids, minocycline, endocannabinoids, and nonsteroidal anti-inflammatory drugs are effective in controlling microglial activation and exert neuroprotective effects in the brain following different types of injuries and neurodegenerative diseases [7, 8]. Although several drugs reduce the symptoms of brain diseases, they are frequently associated with side effects [9, 10]. For the last two decades, the ability of bone marrow-derived mesenchymal stem cells (BM-MSCs) to reduce symptoms of brain diseases such as stroke, Parkinson's disease, and multiple system atrophy has been investigated [11–14].

In addition, BM-MSCs have a therapeutic effect owing to their ability to downregulate inflammatory conditions in the brain [12, 15, 16]. Particularly, the immunomodulatory properties of BM-MSCs play an important role in the treatment of inflammatory diseases, including neurodegenerative disorders. Importantly, this immunomodulatory capacity is highly plastic in response to complex changes in the inflammatory niche. Given the dynamic inflammation in neurodegenerative diseases, BM-MSC-mediated immunomodulation in cell therapy for these diseases deserves more attention [17].

Although many studies have reported the effects of BM-MSC transplantation in animal models of brain disease in reducing neuroinflammation induced by microglia [15, 18, 19], the underlying mechanisms of BM-MSCs in targeting microglia-mediated neuroinflammation and the cellular network of activated microglia are still unclear. In addition, even though mutual reactions between BM-MSC and activated microglia were investigated *in vitro* [20], their interactions were analysed in targeted approaches with limitations to elucidate the corresponding mechanisms. In this study, we evaluated the relationship between rat bone marrow-derived mesenchymal stem cells (rBM-MSCs) and activated microglia using bidirectional transcriptomic analysis.

## 2. Materials and Methods

### 2.1. Isolation and Maintenance of rBM-MSCs

rBM-MSCs were isolated from 8–12-week-old Sprague Dawley (SD) rats and characterised as previously described [21]. Briefly, cells were isolated from the tibias and femurs and separated using 80% Percoll gradient centrifugation at 1300 rpm for 10 min at RT. Cells in the low-density fraction were washed with Dulbecco's modified Eagle's medium (DMEM; Gibco, USA) supplemented with 10% foetal bovine serum (FBS), 100 U/mL penicillin, and 100 *μ*g/mL streptomycin sulphate (HyClone, USA). Next, 1.6 × 10^5^ cells were seeded onto 10 cm culture dishes (SPL, South Korea) containing a control medium and cultured as adherent cells in a humidified chamber at 5% CO_2_ and 37°C for 3–4 weeks. The media was replenished every 3 days. Upon reaching a confluence of 70–80%, cells were subcultured at a ratio of 1 : 4 for up to four passages. We characterised with anti-CD34 monoclonal antibody as negative control and anti-CD29 and CD44 antibodies as positive marker for the characterisation of rBM-MSCs from SD rat. rBM-MSC was highly stained with only positive markers (data not shown), which is consistent with previous report [21].

### 2.2. Rat Microglia Primary Cultures

Microglial primary cultures were obtained from the midbrain of one-day-old Sprague Dawley (SD) rat pups purchased from Nara Bio (Gyeonggi, South Korea). Briefly, mid-brain tissues were isolated from rat pups and rinsed with minimum essential medium (MEM; HyClone, USA) containing 10% FBS, 100 U/mL penicillin, and 100 *μ*g/mL streptomycin sulphate (HyClone, USA). Tissues were mechanically dissociated, and the cells were plated in 10 cm culture dishes (SPL, South Korea). After 13–15 days, the microglia were detached from the flasks and applied to a nylon mesh to remove astrocytes. The collected microglia were cultured in control media in a humidified chamber at 5% CO_2_ and 37°C. Microglia were trypsinized and washed twice with PBS and fixed in Cytofix buffer (BD, USA) for 30 min at room temperature. Cells were incubated 1 h at room temperature with anti-Iba1 mouse monoclonal antibody (1 : 100, Santa Cruz Biotechnology, USA) for characterisation of microglia. Cells were washed twice with PBS and incubated with FITC-conjugated anti-mouse goat antibody (1 : 100, Vector Laboratories, USA) in PBS for 1 h in ice. After washing twice with PBS, labeled cells were analysed by a flow cytometer (FACS Aria II™, BD, USA). Cells showed high expression (~86.5%) of positive marker (Supplementary Figure 1).

### 2.3. Coculture of LPS-Stimulated or Nonstimulated Microglia and rBM-MSCs

To evaluate the effects of LPS-stimulated microglia on rBM-MSCs and of rBM-MSCs on LPS-stimulated microglia, we used a Costar transwell system as previously reported [15], which consists of coculture without direct cell contact between both populations. Subject groups of cells (1–2 × 10^5^ cells/well) were seeded in the bottom chamber, and effector groups of cells (1 × 10^4^ cells/well) were seeded in the insert (0.4 *μ*m pore size; Corning, USA). LPS stimulation of microglia was performed with 100 ng/mL LPS (Sigma, USA) for 4 h prior to coculture. After coculture for 12 h, rBM-MSCs or microglia of subject groups were isolated and processed for further evaluations. Microglial activation was estimated by their morphological differences. Swelled microglia cells with rounder morphology were considered as activated microglia based on criteria for activated microglia [22, 23]. Images were acquired with an optical lens and an Axiovert 200M fluorescence microscope (Zeiss, Jena, Germany).

### 2.4. Transcriptomic Analysis

Differences in gene expression in subject groups of rBM-MSCs or microglia were examined using the Illumina system (Illumina, USA) in conjunction with Sentrix Rat-Ref-12-v1 Expression Bead Chips containing gene-specific oligonucleotides (~22,000 genes, Illumina, USA). Differences in data distribution were analysed using BeadStudio software (Illumina, USA). Probe signals were quantile normalised, and those with a *p* value of less than 0.05 were selected for further analysis. Gene ontology and biological pathways and functions were determined using the web-based bioinformatics software Ingenuity Pathway Analysis (IPA; Ingenuity Systems, USA). A fold change of ±1.2 in expression levels was used as a cut-off to generate data sets of genes with a significantly altered expression. When we applied a stringent cut-off of ±1.5, we cannot compare the groups (experimental and control) and the biological function of the gene network cannot be predicted. This is mainly due to the low abundance of changed genes (Supplementary Figures 2, 3, and 4). Genes with similar molecular functions were grouped and depicted as a network with indicated direct and indirect relationships as previously described [24, 25].

### 2.5. Quantitative Real-Time PCR (qPCR)

The expression levels of genes were quantified with qPCR using SsoAdvanced Universal SYBR Green Supermix real-time PCR kit (Bio-Rad, USA) and cDNA and gene-specific primer pairs (Supplementary Tables 1 and 2) on a Rotor-Gene Q system (Qiagen, USA). Reaction conditions were as follows: 95°C for 5 min, followed by 50 cycles of 95°C for 5 s and 60°C for 30 s. The threshold/quantification cycle (Ct/Cq) value was determined as the point where the detected fluorescence was statistically higher than the background levels. PCR products were analysed using melting curves constructed with Rotor-Gene 1.7 software (Qiagen, USA). PCR reactions were prepared independently in triplicates. Relative quantification of target gene expression was calculated using the 2^−ΔΔCt^ method.

### 2.6. Migration Assay

Migratory activity of rBM-MSCs was determined using an 8 *μ*m pore-size transwell system (Corning, USA). The upper side of the insert was coated with Matrigel (1 : 10 dilution in 0.01 M Tris (pH 8.0), 0.7% NaCl, Corning, USA) for 2 h at 37°C. The bottom chambers contained one of five different conditions for comparison: MEM with 10% FBS as positive control, MEM without FBS as negative control, MEM with 100 ng/mL LPS, 5 × 10^4^ microglia with MEM, and LPS-stimulated 5 × 10^4^ microglia with MEM. Inserts containing 2.5 × 10^4^ rBM-MSCs were overlaid onto each conditioned well and incubated for 12 h. The inserts were carefully washed with cold phosphate-buffered saline (PBS), and nonmigrating cells remaining in the upper side of the inserts were removed with a cotton swab. The insert was fixed in Cytofix buffer (BD, San Jose, CA, USA) at 4°C for 30 min and stained with 10 *μ*g/mL Hoechst 33342 at RT for 10 min. After washing twice with PBS, images were acquired using an Axiovert 200M fluorescence microscope (Zeiss, Jena, Germany). The excitation wavelength for Hoechst 33342 was 405 nm. Migrating cells were counted in ten random 378.27 mm^2^ (710.52 *μ*m × 532.38 *μ*m) microscopic fields using ImageJ software.

### 2.7. Statistical Analysis

Results were analysed using one-way analysis of variance (ANOVA) with Bonferroni's multiple-comparison test as a post hoc test and IBM-SPSS software (IBM, USA). We performed more than three independent experiments and carried out statistical analysis. Differences were considered significant for *p*values < 0.05.

## 3. Results

### 3.1. Cellular Movement-Related Transcriptomic Changes in rBM-MSCs Cocultured with LPS-Stimulated Microglia

**Figure 1:**
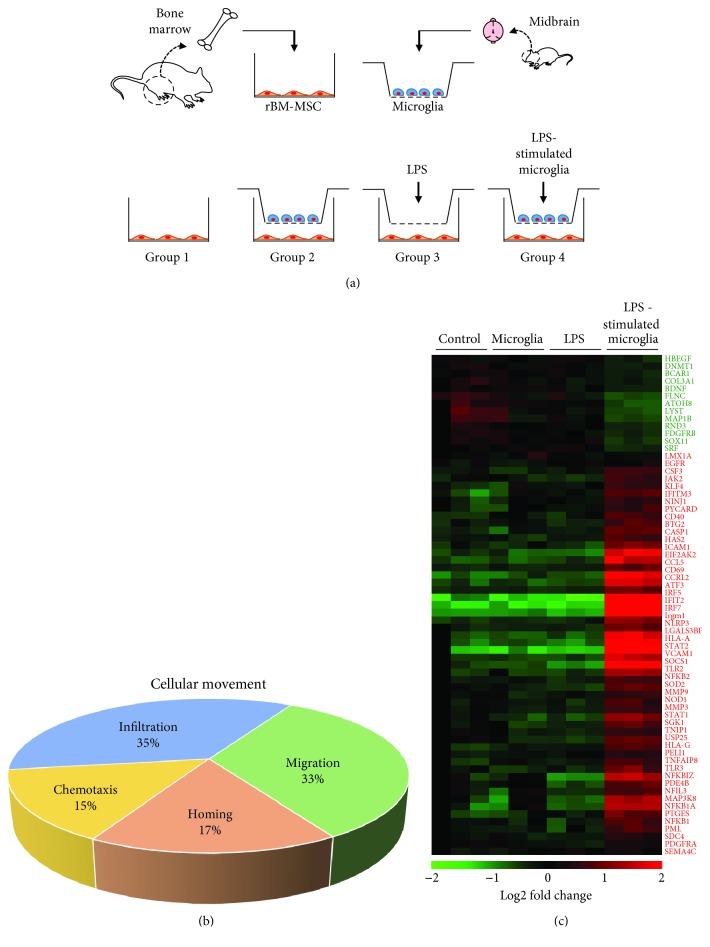
Cellular movement-related gene expression variation in rat bone marrow-derived mesenchymal stem cells (rBM-MSCs) cocultured with LPS-stimulated microglia. (a) *In vitro* coculture experimental design. Four different conditions were used (groups 1–4): group 1, rBM-MSCs only (control); group 2, rBM-MSCs cocultured with microglia; group 3, LPS-treated rBM-MSCs; and group 4, rBM-MSCs cocultured with LPS-stimulated microglia. (b) Gene categorisation according to subgroups related to cellular movement. Genes involved with cellular movement were categorised in subgroups (chemotaxis, homing, migration, and infiltration) based on microarrays and Ingenuity Pathway Analysis. (c) Heat map of genes related to cell migration in the 4 different groups with altered expression levels. Gene expression values are coloured from green (downregulated) to red (upregulated).

To investigate the effects of LPS-stimulated microglia on rBM-MSCs, we used an *in vitro* coculture system (Figure 1(a)). For this study, we isolated rBM-MSCs from 8–12-week-old SD rats and primary microglia from the midbrain of 1-day-old rat pups. Groups of rBM-MSCs were seeded onto the bottom chamber and subjected to 4 different conditions (groups 1–4). Group 1 was the rBM-MSC-only control, group 2 was the coculture with microglia, group 3 was the LPS-treated rBM-MSCs, and group 4 was the coculture with LPS-stimulated microglia. There were no significant changes in cell density of rBM-MSCs (data not shown). In a microarray distribution analysis, gene expression was altered in groups 2, 3, and 4 compared to that of the control (Supplementary Figure 5). In addition, the most pronounced variation in the pattern of gene expression was observed in group 4. Gene ontology analysis of the transcriptome from group 4 revealed that genes from several canonical pathways such as interferon signalling, death receptor signalling, hepatic fibrosis, and neuroinflammation showed variation in expression levels (Supplementary Table 3). Expression of genes related to cellular functions, including death, survival, cell cycle, and cellular movement, was also highly altered in group 4 (Supplementary Figure 6). We focused on the cellular movement function, because the homing of MSCs to the injury site is important for exerting anti-inflammatory effects [26]. For detailed transcriptomic analysis of cellular movement, we selected genes related to cellular movement with altered expression (Figure 1(b)). Heat map analysis showed clear differences in the gene expression pattern in group 4 compared to that of the control (Figure 1(c)).

### 3.2. Functional Prediction of Transcriptomic Networks in rBM-MSCs Cocultured with LPS-Stimulated Microglia

**Figure 2:**
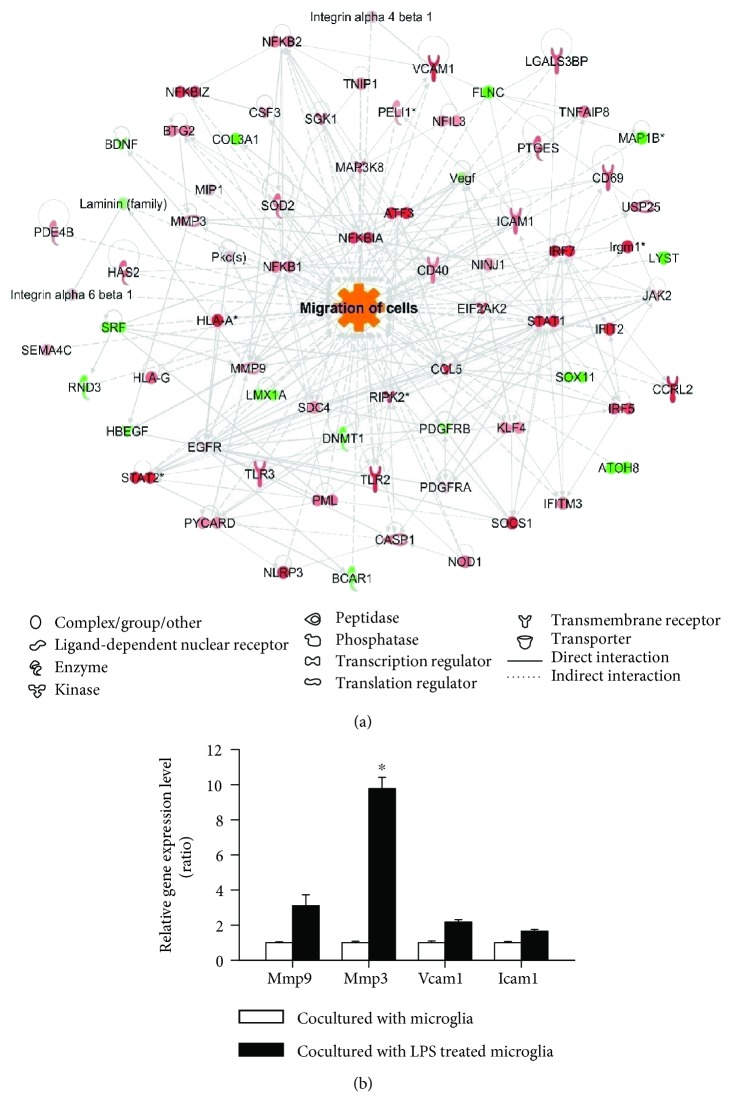
Increase in migration of rat bone marrow-derived mesenchymal stem cells (rBM-MSCs) cocultured with LPS-stimulated microglia. (a) Gene network related to cell migration was constructed, and cellular function was predicted algorithmically using Ingenuity Pathway Analysis. Red and green areas indicate up- and downregulated genes, respectively. Differentially expressed genes were obtained from microarray data (>1.2 fold change). (b) quantitative RT-PCR analysis of gene expression related to cell migration in rBM-MSCs cocultured with LPS-stimulated microglia compared to rBM-MSCs cocultured with microglia. Data represent the mean of three independent experiments. (mean ± SD) ^∗^ *p* < 0.05 versus rBM-MSCs cocultured with microglia.

To obtain detailed information of the genes showing variation in expression, we generated cell movement-related gene expression networks of groups 2, 3, and 4 compared to the network of group 1 using IPA (Supplementary Figure 7). The most pronounced changes in the gene expression networks were also observed in group 4, and the related genes were linked with direct relationships. Based on up- or downregulation and stream relationships, prediction analysis of the networks showed that cell migration is predicted to be activated in group 4 (Figure 2(a)). Four genes with altered expression levels were identified as being highly related to the migratory activity of cells, which was confirmed using quantitative qPCR (Figure 2(b)). The expression levels of matrix metallopeptidases 3 and 9 (Mmp3 and Mmp9, resp.), vascular cell adhesion protein 1 (Vcam1), and intercellular adhesion molecule 1 (Icam1) were significantly higher in group 4 than in group 2.

### 3.3. Increased Migratory Activity in rBM-MSCs Cocultured with LPS-Stimulated Microglia

**Figure 3:**
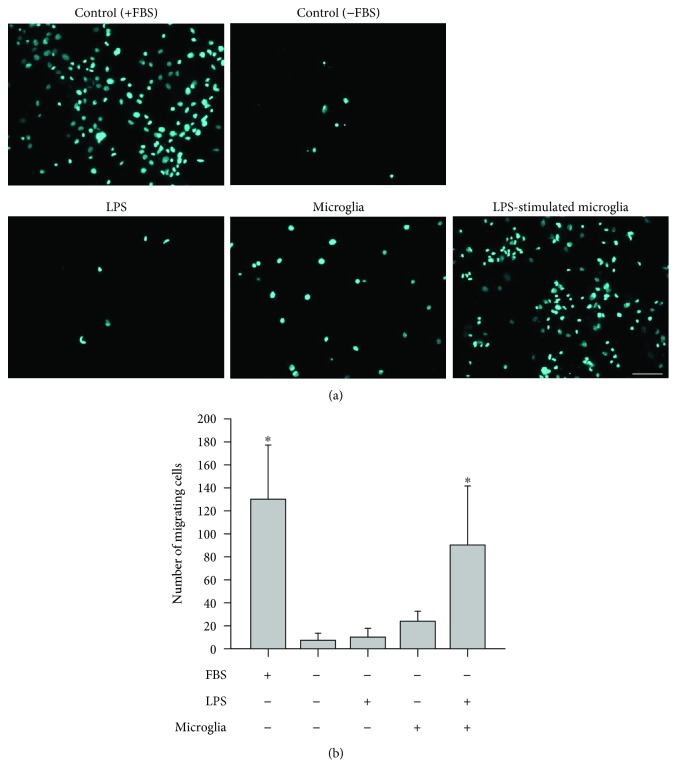
Migration assays with rat bone marrow-derived mesenchymal stem cells (rBM-MSCs) cocultured with LPS-stimulated microglia. (a) Images of migrating rBM-MSCs. Migration of rBM-MSCs was investigated in 5 different conditions: media containing foetal bovine serum (FBS) (positive control), media without FBS (negative control), LPS-treated, cocultured with microglia, and coculture with LPS-stimulated microglia. Migrating cells are cyan in colour (10x magnification). Representative images of migration for each condition are shown. Scale bar: 100 *μ*m. (b) Quantification of migrating cells was performed by counting coloured dots in the images. Data represent the mean of ten random 372.23 mm^2^ (710.52 *μ*m × 532.38 *μ*m) microscopic fields (mean ± SD). ^∗^ *p* < 0.05 versus negative control (media without FBS).

Based on the transcriptomic analysis and prediction, we assumed that there were changes in migratory activity of rBM-MSC influenced by LPS-stimulated microglia. To test the prediction, FBS-containing media condition was used as positive control, and the number of migrating cells was significantly increased in rBM-MSCs when cocultured with LPS-stimulated microglia (Figures 3(a) and 3(b)).

### 3.4. Transcriptomic Analysis in LPS-Stimulated Microglia Cocultured with rBM-MSCs

**Figure 4:**
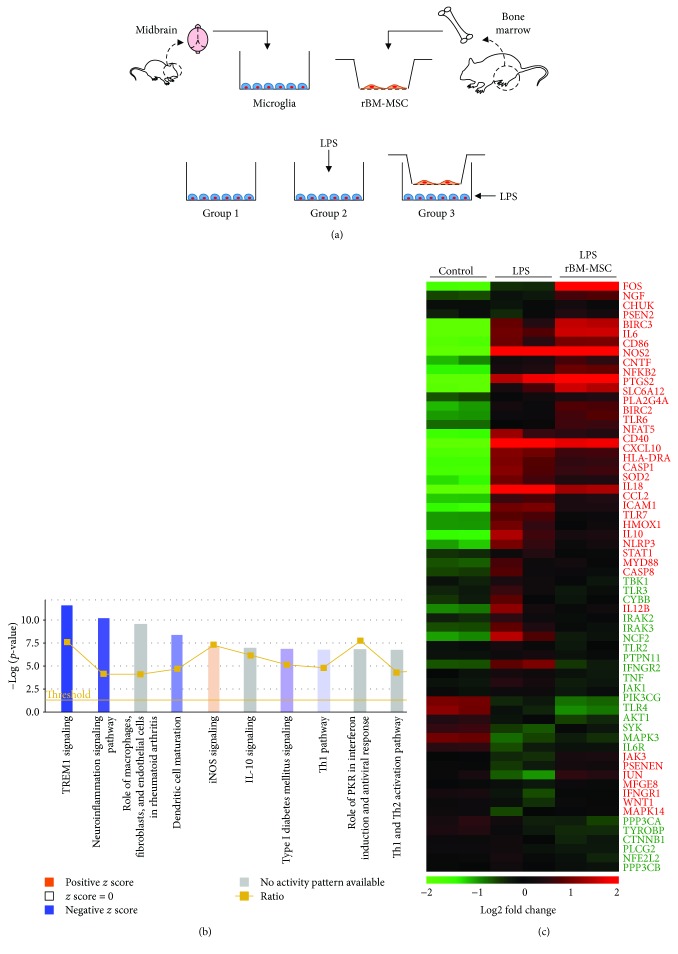
Inflammation-related gene expression variation in LPS-stimulated microglia cocultured with rat bone marrow-derived mesenchymal stem cells (rBM-MSCs). (a) *In vitro* reverse coculture experimental design. Three different conditions were used (group 1–3): group 1, control (microglia only); group 2, LPS-stimulated microglia; and group 3, LPS-stimulation with rBM-MSC coculture. (b) Canonical pathway analysis was constructed algorithmically using Ingenuity Pathway Analysis based on microarray data. Bars indicate canonical pathways containing genes with significantly altered expression. Bar graph colours from blue (inhibition) to orange (activation) represent gene activity of the corresponding pathway according to *z*-score. (c) Heat map of genes related to inflammation with altered expression levels. Gene expression values are coloured from green (downregulated) to red (upregulated).

The effects of rBM-MSCs on LPS-stimulated microglia were also determined via reversed conditions of the aforementioned coculture system *in vitro*. To that end, microglia were seeded on the bottom chamber as subject groups and subjected to 3 different conditions: control, LPS stimulation, and LPS stimulation with rBM-MSC coculture (Figure 4(a)). There were no significant changes in cell density of microglia (data not shown). The transcriptome of subject groups was analysed using microarrays and IPA. Comparison between LPS stimulation and LPS stimulation with rBM-MSC coculture showed changes in expression levels of genes related to cancer, organismal injury and abnormalities, and cell death (Supplementary Figure 8). Gene ontology analysis of the transcriptome revealed altered expression levels of genes related to inflammatory response-related canonical pathways such as triggering receptor expressed on myeloid cell 1 (TREM1) signalling, neuroinflammation, and rheumatoid arthritis (Supplementary Table 4 and Figure 4(b)). Expression of genes related to TREM1 signalling and neuroinflammation was especially suppressed, as predicted (negative *z*-score). Focused gene expression analysis of the inflammatory response showed significantly altered levels in 65 genes between groups (Figure 4(c)). Although the differences were more pronounced after LPS stimulation, remarkable changes were also observed between the presence and absence of rBM-MSCs.

### 3.5. Functional Prediction of Transcriptomic Networks and Reduced Inflammatory Response in LPS-Stimulated Microglia Cocultured with rBM-MSCs

**Figure 5:**
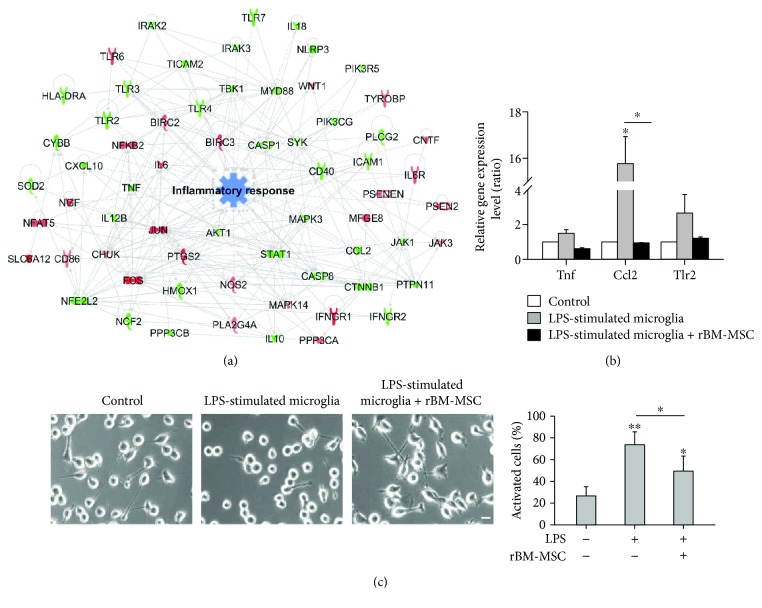
Reduced inflammatory response in LPS-stimulated microglia cocultured with rat bone marrow-derived mesenchymal stem cells (rBM-MSCs). (a) Gene network related to inflammatory response was constructed, and cellular function was predicted algorithmically using Ingenuity Pathway Analysis. Red and green areas indicate up- and downregulated genes, respectively. Differentially expressed genes were obtained from microarray data (>1.2 fold-change). (b) Quantitative real-time PCR analysis of gene expression-related inflammation in LPS-stimulated microglia cocultured with rBM-MSCs compared to control (microglia only). (c) Activated microglia were counted in light microscopy images and quantified as the percentage of activated microglia/total cell number. Cells at the edge of the images were not counted. Scale bar: 20 *μ*m. ^∗^ *p* < 0.05 and ^∗∗^ *p* < 0.01 versus control (microglia only).

To investigate differences in transcriptomes between LPS-stimulated microglia with and without rBM-MSCs, we generated inflammatory response-related gene expression networks using IPA in the corresponding groups (Supplementary Figure 9). A total of 65 genes highly related to the inflammatory response were identified, and the genes were directly linked. Based on the differential expression (upregulation or downregulation), prediction analysis of networks showed that the inflammatory response is predicted to be inhibited by rBM-MSCs (Figure 5(a)). Three genes with altered expression levels were highly related to inflammation, which was confirmed using qPCR (Figure 5(b)). LPS-induced upregulated levels of tumor necrosis factor (Tnf), C-C motif chemokine ligand 2 (Ccl2), and toll-like receptor 2 (Tlr2) genes were significantly decreased after coculture with rBM-MSCs. These predicted functional results were confirmed in experiments using cell cultures, where the number of activated cells induced by LPS stimulation showing swelled and round morphology was significantly decreased by rBM-MSCs (Figure 5(c)). Moreover, the levels of protein markers for microglial activation, CD40 and CD74, were also lower in the presence of rBM-MSC than after LPS stimulation without rBM-MSC, indicating that direct phenotypical activation of microglia was reduced by rBM-MSCs (Supplementary Figure 10).

## 4. Discussion

**Figure 6:**
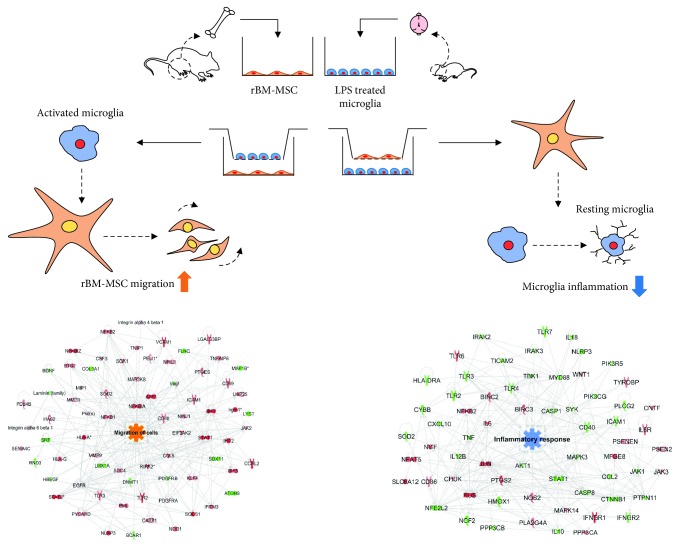
Schematic representation of bidirectional interaction between rat bone marrow-derived mesenchymal stem cells (rBM-MSCs) and activated microglia. Activated microglia increased migration of rBM-MSCs in rBM-MSCs cocultured with LPS-stimulated microglia in an *in vitro* coculture system. rBM-MSCs decreased the inflammatory response of activated microglia in a reverse coculture system.

To the best of our knowledge, this study is the first bidirectional comprehensive investigation of the interaction between activated microglia and MSCs through transcriptomic analysis. We showed that activated microglia increase migration of rBM-MSCs and that rBM-MSCs reduce inflammation of activated microglia at the cellular network level. We analysed the bidirectional cellular interaction that occur between rBM-MSCs and LPS-stimulated microglia using transcriptomics (Figure 6). The results show that (i) LPS-stimulated microglia facilitate homing of rBM-MSCs via inducing transcriptomic changes and that (ii) rBM-MSCs reduce the inflammatory response of microglia by decreasing the expression of the relevant genes.

Transplanted BM-MSCs migrate to the ischaemic border zone in animal models of stroke [27, 28] and to the *substantia nigra* (SN) induced by LPS and MPTP administration in animal models of Parkinson's disease [15], in which inflammatory regions contain activated microglia. In addition, BM-MSCs migrate to LPS-stimulated microglia *in vitro* in response to chemotactic factors [20]. These reports indicate that activated microglia are a possible cause of movement of rBM-MSCs to the site of injury. However, detailed transcriptomic mechanisms of how activated microglia induce the movement of BM-MSCs are not well understood. In this study, we show that the expression levels of 67 genes were significantly altered and that network analysis predicted an increase in migration activity. Meanwhile, CXCR4 receptor and its ligand stromal cell-derived factor-1 (SDF-1a) play an important role in homing of MSCs to brain lesions [29]. However, there were no changes in microarray data and RT-PCR (data not shown). Further studies concerning the homing effects of rBM-MSC on microglia in coculture systems are needed.

The prediction was confirmed experimentally in rBM-MSCs cocultured with LPS-stimulated microglia compared with control and cells cocultured with microglia. Especially, Mmp3 and 9 are essential enzymes for migratory activity and the corresponding genes are induced in inflammatory conditions via proinflammatory cytokines and oxidative substrates [30, 31]. Thus, LPS-stimulated microglia might form a locally conditioned inflammatory region in the coculture system, and rBM-MSCs move toward this region.

It has been reported that human BM-MSCs (hBM-MSCs) dramatically reduce neural damages in a LPS-stimulated mouse brain and reduce inflammation of microglia owing to the production of cytokines and trophic factors [32]. In addition, cocultured BM-MSCs reduce the expression of TNF-alpha and iNOS and NO production in LPS-stimulated microglia compared with noncocultured BM-MSCs [15, 20]. However, the detailed mechanism of how BM-MSCs downregulate activated microglia has not been well studied. In this study, we show that the expression levels of 65 genes were significantly altered, and network analysis predicted the suppression of the inflammatory response. This prediction was experimentally verified in LPS-stimulated microglia cocultured with rBM-MSCs. The results show that the levels of most proinflammatory genes were reduced when cocultured with rBM-MSCs. The expression levels of Ccl2, a key mediator of microglial activation [33, 34], were significantly decreased in the presence of rBM-MSCs, indicating anti-inflammatory effects by rBM-MSC. These phenomena may occur as a result of secreted molecules from both rBM-MSCs and LPS-stimulated microglia. Further studies are needed to identify the secreted molecules, that is, the secretome, that increase invasive migratory activity of rBM-MSCs and decrease the inflammatory response of LPS-stimulated microglia.

There are several limitations to our study. First, we used a fold change of ±1.2 in expression levels as a cut-off and identified low level of abundant genes due to the limitation in an *in vitro* coculture system. In general, the transwell coculture system has fundamental limitations, such as cell density, fluid concentration, and cell-other cell direct interaction, compared to that *in vivo* [35]. However, our transcriptomic analysis for bidirectional effect of each cells and the above approach cannot be done in the *in vivo* system, due to diversity of cells in the organ. We expect that the implied biological effect in our results might be more strongly expressed in *in vivo*. Second, fundamental limitations reside in microarray and bioinformatics analysis. Although microarray is performed with an additional probe for compensation of inaccuracies and statistically analysed, the reduction of false positive or negative is a challenging task [36]. In addition, bioinformatics tool is constructed with existing knowledge, hence novel finding is limited [37]. As a result, a unique pathway cannot be deduced in our analysis system and individual analysis for genes can be biased, which could bring about false positive or negative results. Thus, we focused more in cellular processes than target molecules. Finally, analysing specific pathway/s or molecule/s is beyond our limitation in this study. To find novel pathway/s or molecule/s, responsible for attracting rBM-MSCs or inflammatory response of microglia identified, other methodological biomolecular approaches are required.

In conclusion, we performed bidirectional transcriptomic analysis of activated microglia and BM-MSCs. The results show that activated microglia in a neuroinflammatory condition modulate the migration of BM-MSCs and that BM MSCs reduce inflammatory response in microglia. This study enhances our understanding of the relationship between activated microglia and transplanted stem cells and may lead to a new therapeutic strategy using stem cell therapy for brain diseases.

## Data Availability

The data used to support the findings of this study are available from the corresponding author upon request.

## Supplementary Materials

Supplementary Materials Supplementary Table 1: quantitative RT-PCR primer sequences for genes encoding the transcriptomic network related to cell migration. Supplementary Table 2: quantitative RT-PCR primer sequences for genes encoding the transcriptomic network related to inflammatory response. Supplementary Table 3: top 20 canonical pathways constructed algorithmically by Ingenuity Pathway Analysis in rBM-MSCs cocultured with LPS-stimulated microglia compared to rBM-MSCs cocultured with microglia. Supplementary Table 4: top 20 canonical pathways constructed algorithmically by Ingenuity Pathway Analysis in LPS-stimulated microglia cocultured with rBM-MSCs compared to LPS-stimulated microglia. Supplementary Figure 1: flow cytometry analysis of Iba1-positive cells in primary microglia isolated from SD rat pups. Supplementary Figure 2: transcriptomic network analysis in four different culture conditions of rBM-MSCs (>1.5 fold change). Supplementary Figure 3: gene network related to cell migration in rBM-MSCs cocultured with LPS-stimulated microglia compared to rBM-MSCs cocultured with microglia was constructed algorithmically using Ingenuity Pathway Analysis (>1.5 fold change). Supplementary Figure 4: gene network related to inflammatory response in LPS-stimulated microglia cocultured with rBM-MSCs compared to control (microglia only) was constructed algorithmically using Ingenuity Pathway Analysis (>1.5 fold change). Supplementary Figure 5: plotting of signal intensities in three different culture conditions of rBM-MSCs compared to control (rBM-MSCs only). Supplementary Figure 6: top 20 list of function or diseases constructed algorithmically by Ingenuity Pathway Analysis in rBM-MSCs cocultured with LPS-stimulated microglia compared to rBM-MSCs cocultured with microglia. Supplementary Figure 7: transcriptomic network analysis in four different culture conditions of rBM-MSCs (>1.2 fold change). Supplementary Figure 8: top 20 list of function or diseases constructed algorithmically by Ingenuity Pathway Analysis in LPS-stimulated microglia cocultured with rBM-MSCs compared to LPS-stimulated microglia. Supplementary Figure 9: transcriptomic network analysis in two different culture conditions of LPS-stimulated microglia (>1.2 fold change). Supplementary Figure 10: Western blot analysis in LPS-stimulated microglia cocultured with rBM-MSCs using anti CD40 and CD74 antibodies.
