# Supplementary material for: Bidirectional Transcriptome Analysis of Rat Bone Marrow-Derived Mesenchymal Stem Cells and Activated Microglia in an In Vitro Coculture System
Source: Stem Cells Int. 2018 Jul 29;2018:6126413. doi: 10.1155/2018/6126413 (PMC6087576; doi:10.1155/2018/6126413)
Supplement: Supplementary Materials — Supplementary Table 1: quantitative RT-PCR primer sequences for genes encoding the transcriptomic network related to cell migration. Supplementary Table 2: quantitative RT-PCR primer sequences for genes encoding the transcriptomic network related to inflammatory response. Supplementary Table 3: top 20 canonical pathways constructed algorithmically by Ingenuity Pathway Analysis in rBM-MSCs cocultured with LPS-stimulated microglia compared to rBM-MSCs cocultured with microglia. Supplementary Table 4: top 20 canonical pathways constructed algorithmically by Ingenuity Pathway Analysis in LPS-stimulated microglia cocultured with rBM-MSCs compared to LPS-stimulated microglia. Supplementary Figure 1: flow cytometry analysis of Iba1-positive cells in primary microglia isolated from SD rat pups. Supplementary Figure 2: transcriptomic network analysis in four different culture conditions of rBM-MSCs (>1.5 fold change). Supplementary Figure 3: gene network related to cell migration in rBM-MSCs cocultured with LPS-stimulated microglia compared to rBM-MSCs cocultured with microglia was constructed algorithmically using Ingenuity Pathway Analysis (>1.5 fold change). Supplementary Figure 4: gene network related to inflammatory response in LPS-stimulated microglia cocultured with rBM-MSCs compared to control (microglia only) was constructed algorithmically using Ingenuity Pathway Analysis (>1.5 fold change). Supplementary Figure 5: plotting of signal intensities in three different culture conditions of rBM-MSCs compared to control (rBM-MSCs only). Supplementary Figure 6: top 20 list of function or diseases constructed algorithmically by Ingenuity Pathway Analysis in rBM-MSCs cocultured with LPS-stimulated microglia compared to rBM-MSCs cocultured with microglia. Supplementary Figure 7: transcriptomic network analysis in four different culture conditions of rBM-MSCs (>1.2 fold change). Supplementary Figure 8: top 20 list of function or diseases constructed algorithmically by Ingenui [file 6126413.f1.pdf]

Supporting Information

Bidirectional transcriptome analysis of rat bone marrow-derived mesenchymal stem cells and activated microglia in an *in vitro* co-culture system

Da Yeon Lee[1], Moon Suk Jin[1], Balachandran Manavalan[1], Hak Kyun Kim[2], Jun Hyeok Song[2], Tae Hwan Shin[1,3]★★, and Gwang Lee[1,3]★

[1]*Department of Physiology and Department of Biomedical Sciences, Ajou University School of Medicine, Suwon, Republic of Korea*

[2]*Ajou University School of Medicine, Suwon, Republic of Korea*

[3] *Institute of Molecular Science and Technology, Ajou University, Suwon, Republic of Korea*

★★Co-corresponding author: Tae Hwan Shin

Research fellow, Department of Physiology

Ajou University School of Medicine, 164, World cup-ro

Yeongtong-gu, Suwon, 16499, Republic of Korea.

Tel: +82-31-219-4555

Fax: +82-31-219-5049

E-mail: catholicon@ajou.ac.kr

★Corresponding author: Gwang Lee

Professor, Department of Physiology

Ajou University School of Medicine, 164, World cup-ro,

Yeongtong-gu, Suwon, 16499, Republic of Korea.

Tel: +82-31-219-4554

Fax: +82-31-219-5049

E-mail: glee@ajou.ac.kr

Supplementary Table 1: Quantitative RT-PCR primer sequences for genes encoding transcriptomic network related to cell migration

Gene Name	Symbol	NCBI Ref. seq	Direction	Primer sequence (5'-3')
Rattus norvegicus intercellular adhesion molecule 1	Icam1	NM_012967.1	Forward	ACA GCA TTT ACC CCT CAC C
			Reverse	CAG GTC ACG AGT TCA CAG TC
Rattus norvegicus vascular cell adhesion molecule 1	Vcam1	NM_012889.1	Forward	TCC AAC TCC CAA AAT CCT GTG
			Reverse	TTC CAG CCT CAT TAA TCC CTT C
Rattus norvegicus matrix metallopeptidase 9	Mmp9	NM_031055.1	Forward	TCA CTT TCC CTT CAC CTT CG
			Reverse	CTC CGT GTA GAG ATT CTC ACT G
Rattus norvegicus matrix metallopeptidase 3	Mmp3	NM_133523.3	Forward	TCT TCC TCT GAA ACT TGG CG
			Reverse	AGT GCT TCT GAA TGT CCT TCG

Ref. seq.: Reference sequence

Supplementary Table 2: Quantitative RT-PCR primer sequences for genes encoding transcriptomic network related to inflammatory response

Gene Name	Symbol	NCBI Ref. seq	Direction	Primer sequence (5'-3')
Rattus norvegicus toll-like receptor 2	Tlr2	NM_198769.2	Forward	GGA TCT TGA TGG CTG TGA TAG G
			Reverse	CTT TGT GTT TGC TGT GAG TCC
Rattus norvegicus C-C motif chemokine ligand 2	Ccl2	NM_031530.1	Forward	GGA CTT CAG CAC CTT TGA ATG
			Reverse	GGA CTT CAG CAC CTT TGA ATG
Rattus norvegicus tumor necrosis factor	Tnf	NM_012675.3	Forward	TGG GCT GTA CCT TAT CTA CTC C
			Reverse	GGC TGA CTT TCT CCT GGT ATG
Rattus norvegicus glyceraldehyde-3-phosphate dehydrogenase	Gapdh	NM_017008.4	Forward	GAA GAC TGT GGA TGG CCC
			Reverse	CCA TGC CAG TGA GCT TCC

Ref. seq.: Reference sequence

Supplementary Table 3: Top 20 **c**anonical pathways constructed algorithmically by Ingenuity Pathway Analysis in rBM-MSCs co-cultured with LPS-stimulated microglia compared to rBM-MSCs co-cultured with microglia

**Canonical Pathways**	**-log(p-value)**	**Number of genes**
Interferon Signaling	13.7	20
Death Receptor Signaling	10.6	28
Hepatic Fibrosis / Hepatic Stellate Cell Activation	9.1	39
Neuroinflammation Signaling Pathway	8.71	53
iNOS Signaling	8.38	17
Activation of IRF by Cytosolic Pattern Recognition Receptors	8.21	20
TREM1 Signaling	7.51	21
Protein Ubiquitination Pathway	7.01	44
PPAR Signaling	6.87	23
Apoptosis Signaling	6.68	22
Role of JAK1, JAK2 and TYK2 in Interferon Signaling	6.65	11
Osteoarthritis Pathway	6.55	37
Granulocyte Adhesion and Diapedesis	6.47	33
TNFR2 Signaling	6.41	12
Role of Macrophages, Fibroblasts and Endothelial Cells in Rheumatoid Arthritis	6.15	47
Colorectal Cancer Metastasis Signaling	6.09	49
IL-10 Signaling	6.03	18
Role of Pattern Recognition Receptors in Recognition of Bacteria and Viruses	6	27
TNFR1 Signaling	5.96	15
Hepatic Cholestasis	5.61	29

Supplementary Table 4: Top 20 canonical pathways constructed algorithmically by Ingenuity Pathway Analysis in LPS-stimulated microglia co-cultured with rBM-MSCs compared to LPS-stimulated microglia

Canonical Pathways	-log(p-value)	Number of genes
TREM1 Signaling	11.7	31
Neuroinflammation Signaling Pathway	10.4	73
Role of Macrophages, Fibroblasts and Endothelial Cells in Rheumatoid Arthritis	9.65	70
Dendritic Cell Maturation	8.54	49
iNOS Signaling	7.17	19
IL-10 Signaling	7.04	24
Type I Diabetes Mellitus Signaling	6.93	31
Th1 Pathway	6.87	35
Role of PKR in Interferon Induction and Antiviral Response	6.83	18
Th1 and Th2 Activation Pathway	6.82	43
PI3K Signaling in B Lymphocytes	6.45	34
Altered T Cell and B Cell Signaling in Rheumatoid Arthritis	6.31	26
Colorectal Cancer Metastasis Signaling	5.97	51
JAK/Stat Signaling	5.88	24
Role of Pattern Recognition Receptors in Recognition of Bacteria and Viruses	5.81	33
Crosstalk between Dendritic Cells and Natural Killer Cells	5.75	25
HMGB1 Signaling	5.64	32
Production of Nitric Oxide and Reactive Oxygen Species in Macrophages	5.51	42
Death Receptor Signaling	5.46	25
Toll-like Receptor Signaling	5.46	22

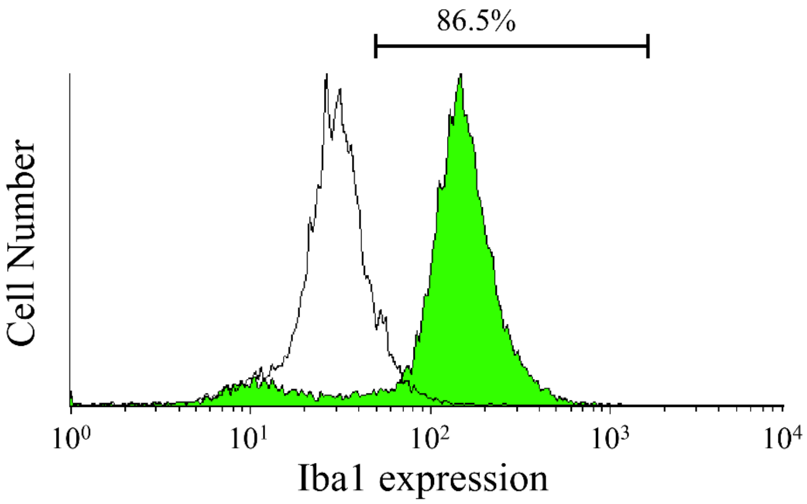

Supplementary Figure 1: Flow cytometry analysis of Iba1 positive cells in primary microglia isolated from SD rat pups. Flow cytometry analysis was performed by staining with anti-Iba1. The percentage of Iba1 positive cells were determined as purity of microglia.

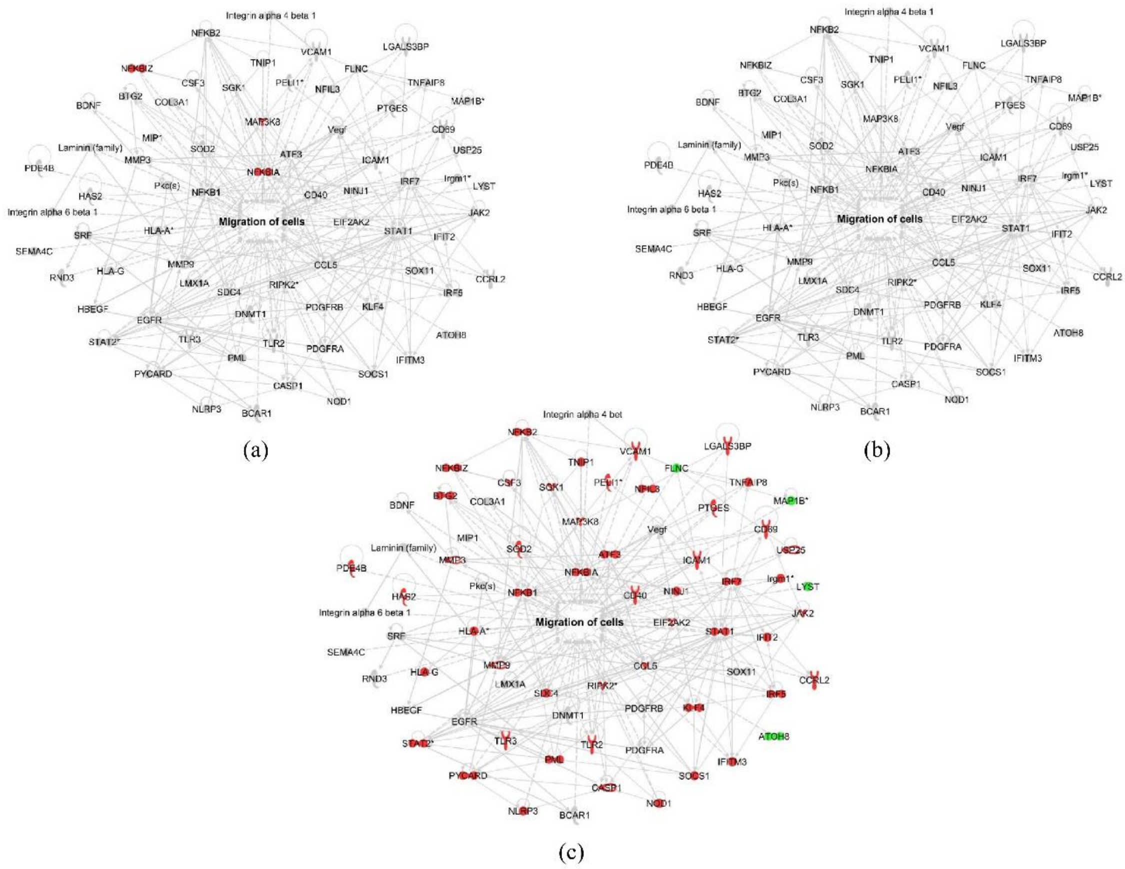

Supplementary Figure 2: Transcriptomic network analysis in four different culture conditions of rBM-MSCs. Gene network related to cell migration was constructed algorithmically using Ingenuity Pathway Analysis. Transcriptome network of (a) rBM-MSCs co-cultured with microglia, (b) LPS-treated rBM-MSCs, and (c) rBM-MSCs co-cultured with LPS-stimulated microglia compared to control (rBM-MSCs only), respectively were shown. Red and green areas indicate up- and down-regulated genes, respectively. Differentially expressed genes were obtained from microarray data (>1.5 fold-change).

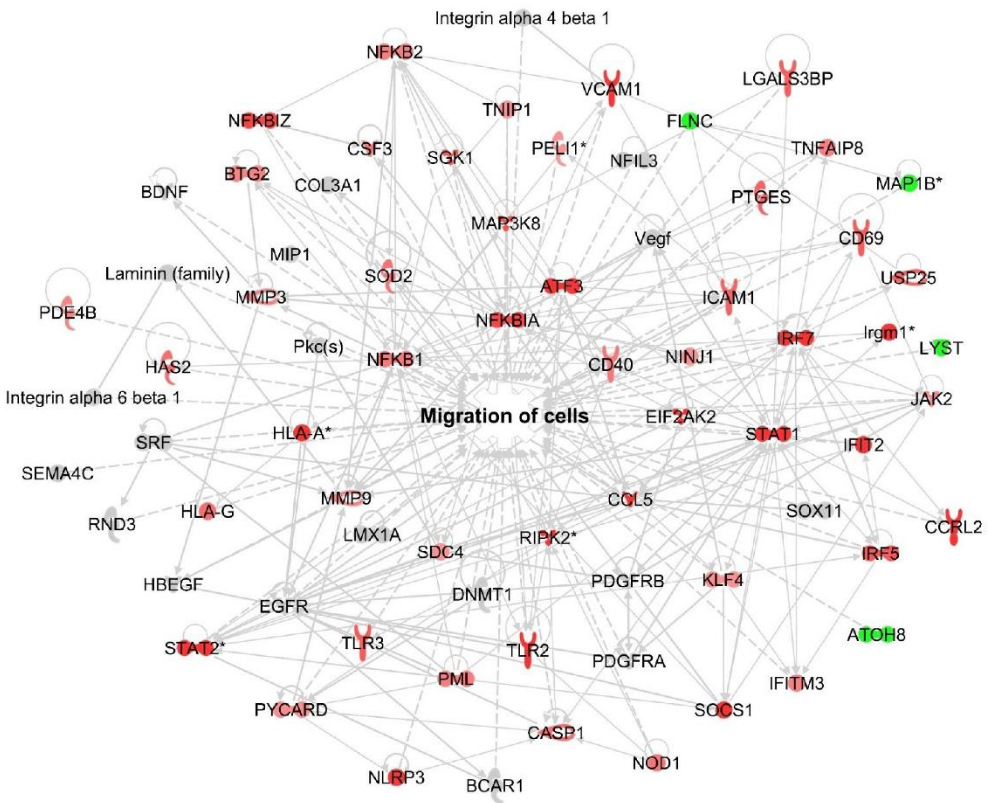

Supplementary Figure 3: Gene network related to cell migration in rBM-MSCs co-cultured with LPS-stimulated microglia compared to rBM-MSCs co-cultured with microglia was constructed algorithmically using Ingenuity Pathway Analysis. Red and green areas indicate up- and down-regulated genes, respectively. Differentially expressed genes were obtained from microarray data (>1.5 fold-change).

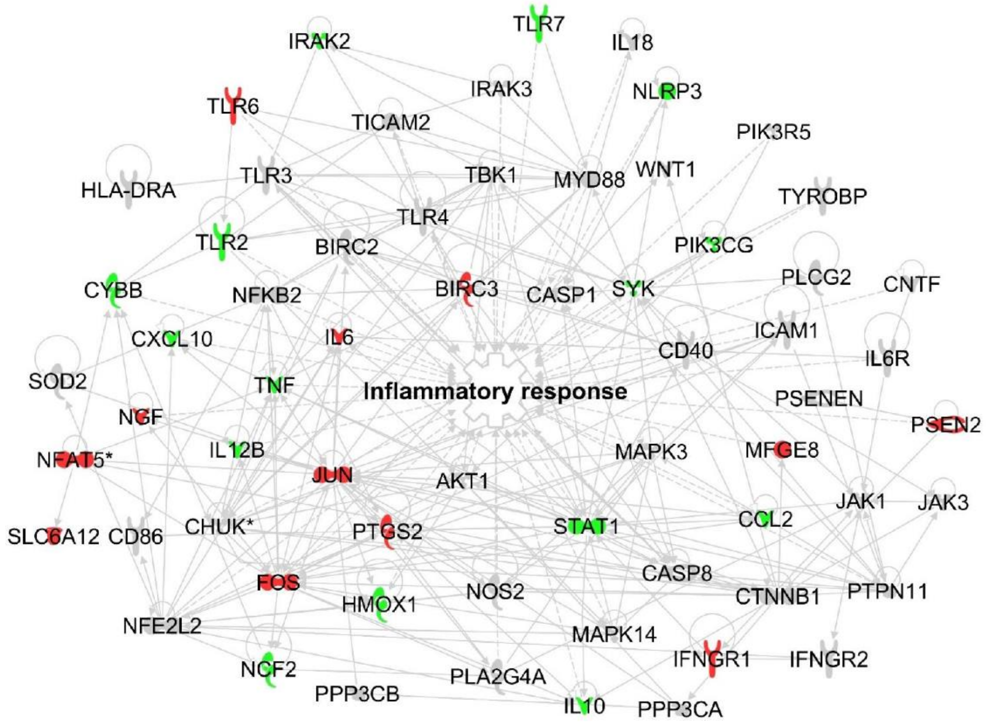

Supplementary Figure 4: Gene network related to inflammatory response in LPS-stimulated microglia co-cultured with rBM-MSCs compared to control (microglia only) was constructed algorithmically using Ingenuity Pathway Analysis. Red and green areas indicate up- and down-regulated genes, respectively. Differentially expressed genes were obtained from microarray data (>1.5 fold-change).

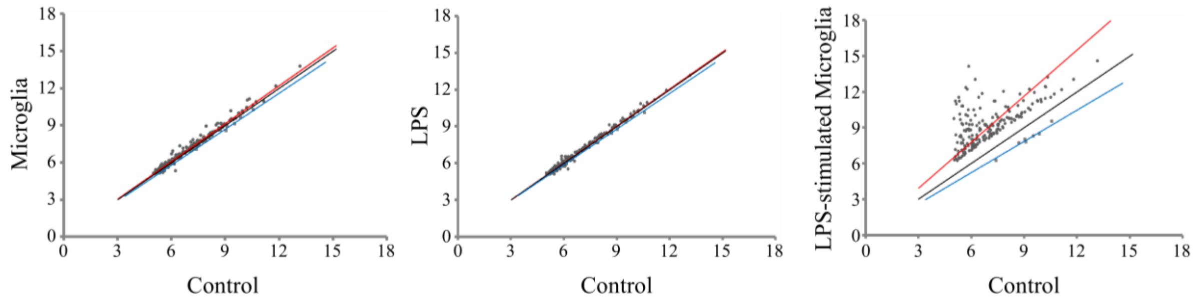

(a) (b) (c)

Supplementary Figure 5: Plotting of signal intensities in three different culture conditions of rBM-MSCs compared to control (rBM-MSCs only). Correlation of gene expression variation in experimental groups. The X axes indicate the signal intensities of control and Y axes indicate (a) rBM-MSCs co-cultured with microglia, (b) LPS-treated rBM-MSCs, and (c) rBM-MSCs co-cultured with LPS-stimulated microglia, respectively. The black line indicates the criterion line which is standardized with control intensities.

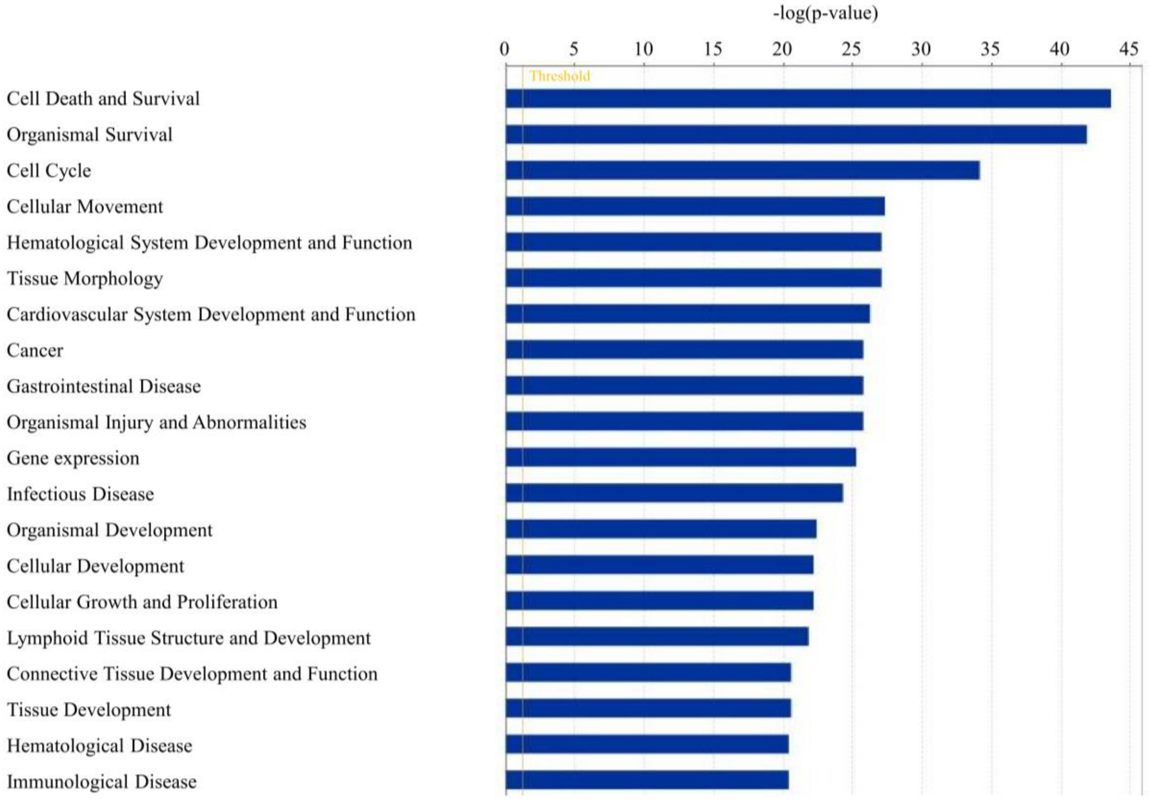

Supplementary Figure 6: Top 20 list of function or diseases constructed algorithmically by Ingenuity Pathway Analysis in rBM-MSCs co-cultured with LPS-stimulated microglia compared to rBM-MSCs co-cultured with microglia.

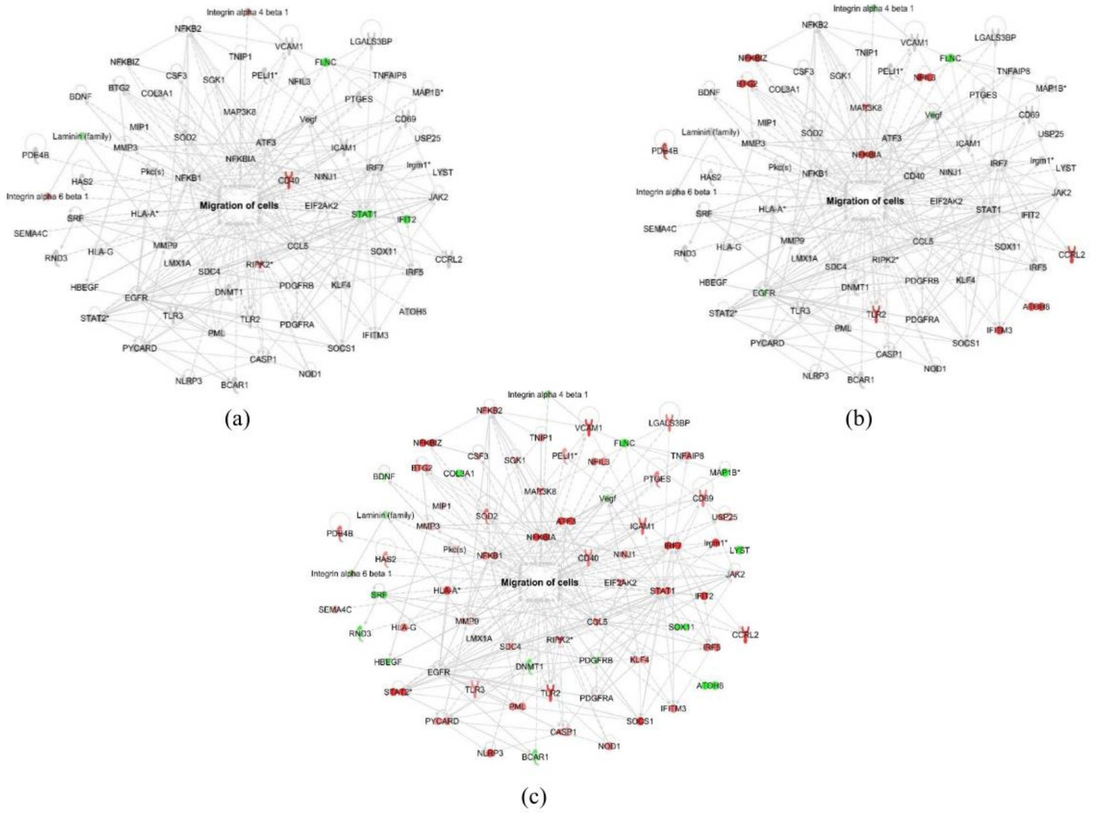

Supplementary Figure 7: Transcriptomic network analysis in four different culture conditions of rBM-MSCs. Gene network related to cell migration was constructed algorithmically using Ingenuity Pathway Analysis. Transcriptome network of (a) rBM-MSCs co-cultured with microglia, (b) LPS-treated rBM-MSCs, and (c) rBM-MSCs co-cultured with LPS-stimulated microglia compared to control (rBM-MSCs only), respectively were shown. Red and green areas indicate up- and down-regulated genes, respectively. Differentially expressed genes were obtained from microarray data (>1.2 fold-change).

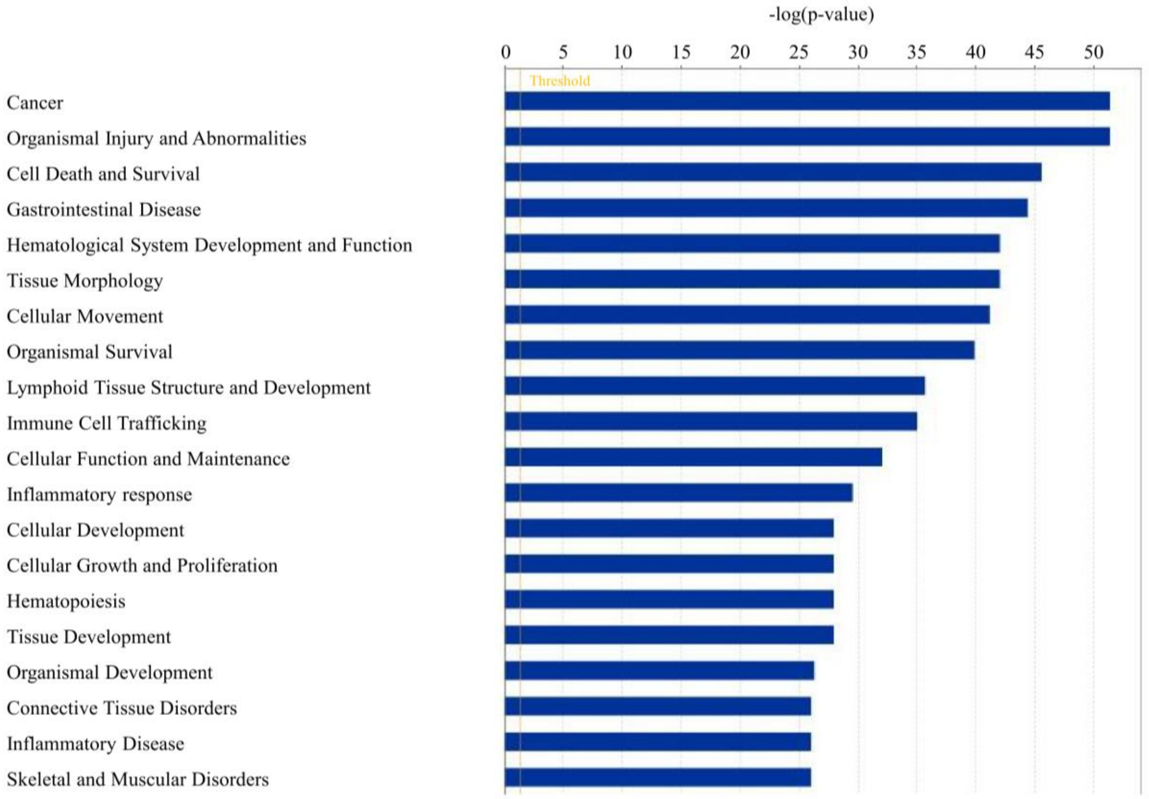

Supplementary Figure 8: Top 20 list of function or diseases constructed algorithmically by Ingenuity Pathway Analysis in LPS-stimulated microglia co-cultured with rBM-MSCs compared to LPS-stimulated microglia.

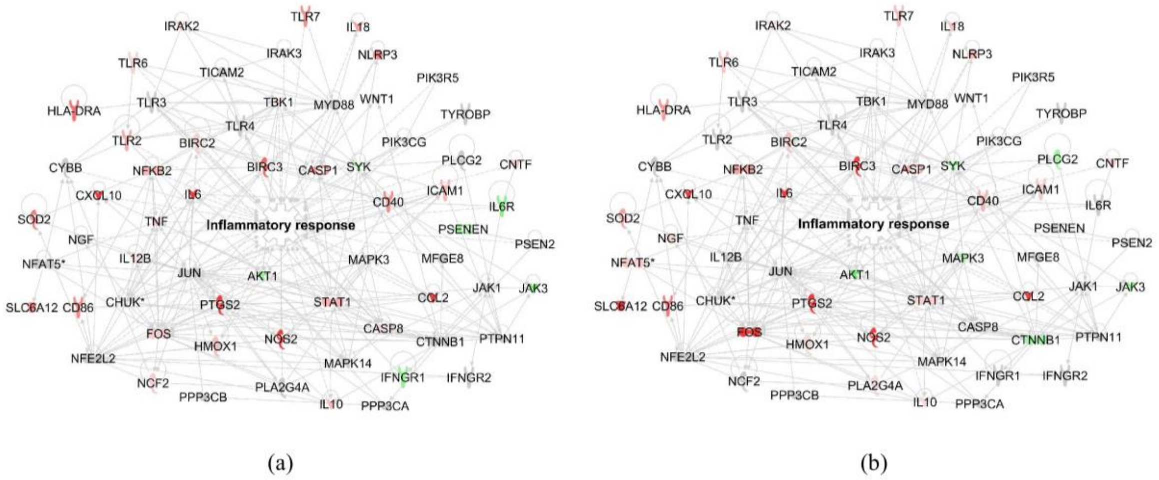

Supplementary Figure 9: Transcriptomic network analysis in two different culture conditions of LPS-stimulated microglia. Gene network related to inflammatory response was constructed algorithmically using Ingenuity Pathway Analysis. Transcriptome network of (a) LPS-stimulated microglia and (b) LPS-stimulated microglia co-cultured with rBM-MSCs compared to control (microglia only), respectively were shown. Red and green areas indicate up- and down-regulated genes, respectively. Differentially expressed genes were obtained from microarray data (>1.2 fold-change).

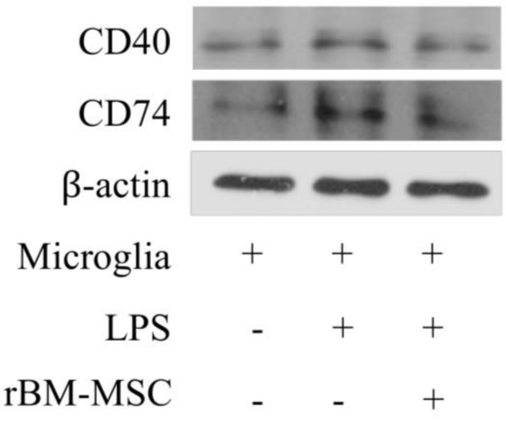

Supplementary Figure 10: Western blot analysis in LPS-stimulated microglia co-cultured with rBM-MSCs. Total Protein extracted from microglia in three different conditions. Samples were resolved on a 15% gel and western blotting was performed using anti CD40 and CD74 antibodies. β-actin served as a loading control.
